# Toward Extensive Utilization of Pulping Liquor from Chemical–Mechanical Pulping Process of Wheat Straw in Biorefinery View

**DOI:** 10.3390/molecules29225368

**Published:** 2024-11-14

**Authors:** Ning Sun, Xingxiang Ji, Zhongjian Tian, Baobin Wang

**Affiliations:** 1 School of Bionengneering, Qilu University of Technology, Jinan 250013, China; 17864170532@163.com; 2 State Key Laboratory of Biobased Material & Green Papermaking, Qilu University of Technology, Jinan 250013, China; xxjt78@163.com (X.J.); baobinqlu@qlu.edu.cn (B.W.)

**Keywords:** wheat straw, chemical–mechanical pulp, acid treatment, lignin, xylo-oligosaccharides (XOSs)

## Abstract

Extensive utilization of renewable biomass is crucial for the progress of carbon neutral and carbon peak implementation. Wheat straw, as an important by-product of crops, is hardly ever efficiently utilized by conventional processes. Here, we proposed a mild acid-coupled-with-enzymatic-treatment process to realize the utilization of lignin and hemicelluloses from pulping liquor on the basis of the chemical–mechanical pulping process. The pulping liquor was treated with acid first to precipitate lignin, and it was further hydrolyzed with xylanase to obtain XOSs. The recovered lignin was characterized by FT-IR, 2D-HSQC, GPC, etc. It was found that lignin undergoes depolymerization and condensation during acid treatment. Also, saccharide loss enhanced with the decrease in pH due to the presence of the LCC structure. As a result, an optimized pH of 4 for the acid treatment ensured that the removal rate of lignin and loss rate of polysaccharides achieved 77.15% and 6.13%, respectively. Moreover, further xylanase treatment of the pulping liquor attained a recovery rate of 51.87% for XOSs. The study presents a new insight for the efficient utilization of lignin and hemicellulose products from non-woody materials in the prevailing biorefinery concept.

## 1. Introduction

Extensive utilization of renewable biomass is crucial for the progress of carbon neutral and carbon peak implementation [1]. Woody biomasses utilize biorefinery concepts such as the prehydrolysis-based kraft pulping process (PHK) to achieve separation and utilization of the main constituents of lignocellulosic biomass [2,3]. However, wheat straw, as a supplement resource of woody biomass [4], annually regenerates around 143 million tons in North China, and it can hardly be utilized by conventional chemical pulping processes due to silicon interference [5], and the prevailing strategies including combustion and direct returning to fields lead to potential environmental pollution and soil compaction. Therefore, it is critical to utilize wheat straw in a facile and sustainable manner.

High-yield pulping process is combined chemical and mechanical treatment to disintegrate biomass into cellulose fibrils, and high-yield pulp accounts for a great portion during the production of board and paper products [6]. Our group proposed a facile enzyme-facilitated chemical–mechanical pulping process which improved the pulp property, and decreased energy cost and pulping liquor discharge, thus enhancing the potential utilization of wheat straw [7,8]. Due to the sustainable process, over 70% of wheat straw is converted to cellulose pulp, and around 14% of the components is degraded into the pulping liquor. The degradation components are under-utilized in the conventional treatment process which requires vast energy and chemical input to eliminate the potential environmental risk. There is great potential for the value-added utilization of by-products such as lignin nanoparticles and xylo-oligosaccharides (XOSs) [9,10]. Until now, it is still a challenge to separate and utilize the degraded resources in the pulping liquor. As far as we know, there are no reports on utilizing degraded resources in the pulping liquor.

Acid precipitation method for lignin separation is highly efficient, easy to operate and inexpensive, which is the main strategy for modern industrialized lignin. Acid treatment is normally utilized in acid isolation of soda lignin from the black liquor treatment of the chemical pulping process, and the current pulping liquor of chemical–mechanical pulp undergoes a wastewater treatment process which leads to a great amount of chemical cost and environmental burden [11,12]. The process we proposed in this manuscript presents a novel strategy for the recovery of lignin and hemicelluloses, which are the main components in the pulping liquor. Additionally, acid treatment is a well-established stage in practical industrial fields and is feasible to realize the efficient separation of lignin and hemicelluloses from pulping liquor, thus favoring the further hydrolysis of hemicelluloses. Also, the subsequent enzymatic process does not require additional acid, thus reducing the amount of acid neutralization and acid usage. Therefore, lignin is separated and the pollution load is effectively reduced.

In the current pulp and paper industry, the pulping liquor produced by chemical pulp is mostly treated as effluent, which results in a waste of biomass resources and environmental burden. Due to the chemical and mechanical treatment of chemical–mechanical pulping of wheat straw, the main components in the pulping liquor are lignin derivatives and hemicellulose-derived saccharide oligomers [13]. The lignin derivatives are in the colloidal state due to the unique physiochemical properties, and the saccharide oligomers are dissolved in the pulping liquor. Similarly, the commercialized process utilized the acid precipitation process to recover kraft lignin in the chemical pulping process [14,15]. However, the process inevitably leads to saccharide loss. In view of this, an acid-induced protonation process is introduced by leveraging lignin separation efficiency and saccharide loss of the pulping liquor. The mechanism of lignin removal was verified by different characterization techniques including Zeta potential analysis, particle size analysis, UV-vis spectra, FTIR, NMR, GPC, etc. The treated pulping liquor is further treated with enzymes to obtain a high yield of XOSs. The combined acid treatment and enzyme process proposed in this paper not only realized the recovery of lignin and hemicelluloses, but also reduced the pollution load and external cost for pulping industries, which is of great significance to the development of biomass-refining technology [16].

## 2. Results and Discussion

### 2.1. Pulping Liquor Extensive Utilization Scheme of the Chemical–Mechanical Pulping Process of Wheat Straw

**Figure 1:**
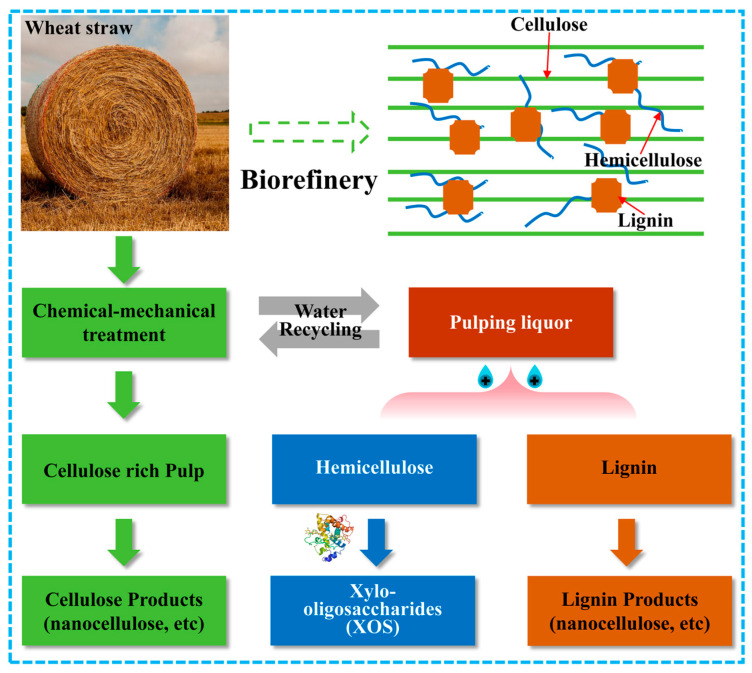
Pulping liquor extensive utilization scheme of the chemical–mechanical pulping process of wheat straw.

Wheat straw, a kind of renewable non-woody biomass, has drawn great attention as feedstock for pulp industry and biorefinery. However, conventional chemical pulping processes and ethanol production technology have suffered from issues such as chemical recycling and high operation costs [17]. Thus, our group previously proposed a modified chemical–mechanical pulping strategy which utilizes chemical and mechanical processes to disintegrate the integrity of wheat straw into high-yield pulp, as shown in Figure 1. The obtained cellulose-rich pulp can be utilized further for cellulose product such as nanocellulose, cellulose derivatives and paper products [18,19]. Herein, the degraded products mainly derived from lignin and hemicellulose in the pulping liquor achieved facile separation via the protonation of lignin. The lignin can be further utilized to prepare functional lignin materials such as nanolignin [20]. The rest stream which contains mainly hemicelluloses was further valorized by enzymatic hydrolysis to obtain xylo-oligosaccharides (XOSs). This strategy presents a facile strategy to realize the extensive utilization of wheat straw based on the mild chemical–mechanical pulping process which would enlighten the valorization of other non-woody biomass materials.

### 2.2. Facile Separation of Lignin and Saccharides of the Pulping Liquor

**Figure 2:**
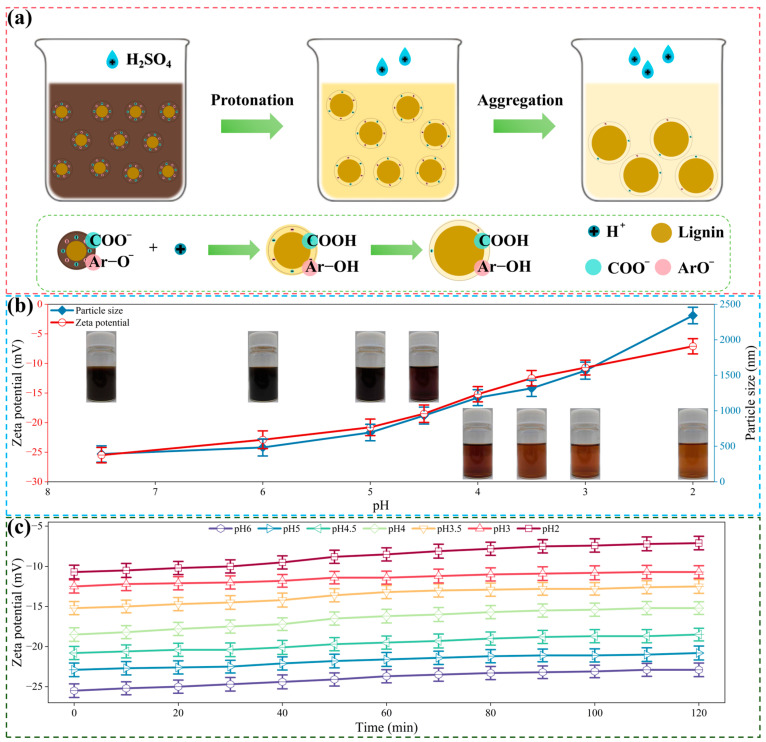
Facile separation of lignin and saccharides of the pulping liquor. (**a**) Mechanism of lignin removal in the pulping liquor during acid treatment. (**b**) Zeta potential and particle size variation in the pulping liquor during acid treatment (the inset figures are the supernatant of the pulping liquor after acid treatment under corresponding pH). (**c**) Zeta potential variation in the pulping liquor upon different treatment times during acid treatment.

**Figure 3:**
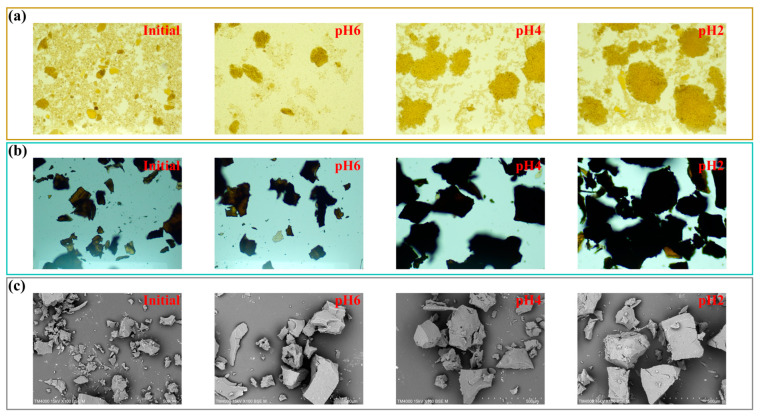
Optical microscopy and SEM images of pulping liquor at different pH levels. (**a**) Optical microscopic morphology of pulping liquor samples at different pH levels. (**b**) Optical microscopic morphology of lignin samples under different pH conditions. (**c**) Scanning electron microscopy morphology of lignin samples under different pH conditions.

Conventional pulping liquor utilizes wastewater treatment to reduce the environmental risk which leads to a high operation cost and waste of resources [21]. Herein, we utilized the commercially available process to recover lignin and hemicelluloses in the pulping liquor to realize the extensive utilization of wheat straw, as shown in Figure 2a. The pulping liquor was treated with a modified acid process to induce the precipitation of lignin, and the hemicellulose-degraded saccharides can thus be recovered. As can be seen in Figure 2b, the Zeta potential increased from −25.5 mV to −7.1 mV as the pH decreased from the initial value to 2. The inset pictures demonstrated that the supernatant of the pulping liquor after acid precipitation becomes transparent and light in color with the decrease in pH treatment after centrifugation, indicating the removal of lignin-degraded products. Moreover, the Zeta potential at different pH values showed a slow increasing trend with the increase in treatment time, and the variation in Zeta potential is significant within the range of 30 to 60 min (Figure 2c). This is due to the ionic moieties presented in the pulping liquor; thus, the lignin clusters can be neutralized, aggregated and finally precipitated [22]. The negative charge of pulping liquor increased with the acid treatment process which is due to the protonation of carboxylic and phenolic groups of lignin. The lignin-degraded products would aggregate via π-π interaction [23], and the dissolved lignin are thus precipitated from the pulping liquor. Meanwhile, the hydrodynamic size of lignin clusters increased from 391 nm to 2342 nm with the increase in the Zeta potential. The lignin clusters would be neutralized, inducing the aggregation of lignin in the pulping liquor, leading to the enhancement in size of lignin clusters. As can be seen in Figure 3, as the pH decreased from the initial (7.5) value to 2, the microscopy and SEM images showed enhanced lignin particles, which is related to the aggregation of lignin clusters.

Generally, lignin carbohydrate complex (LCC), which is cleaved from the chemical and mechanical pulping process, existed in the pulping liquor [24]. Therefore, the hemicellulose-related product would inevitably be removed during the acid treatment process (Table 1). The lignin and polysaccharides in the pulping liquor decreased with the decrease in pH levels. This is because the LCC structure precipitates with lignin during acid treatment in the pulping liquor, resulting in a decrease in sugar content in the supernatant [25]. Also, the acetic acid content in the pulping liquor gradually increases as the pH value decreases, while the furfural and 5-HMF contents showed minimal changes. The increase in acetic acid content may be due to the cleavage of acetyl groups attached to hemicellulose under acidic conditions, which further verified the existence of LCC in the pulping liquor [26].

**Figure 4:**
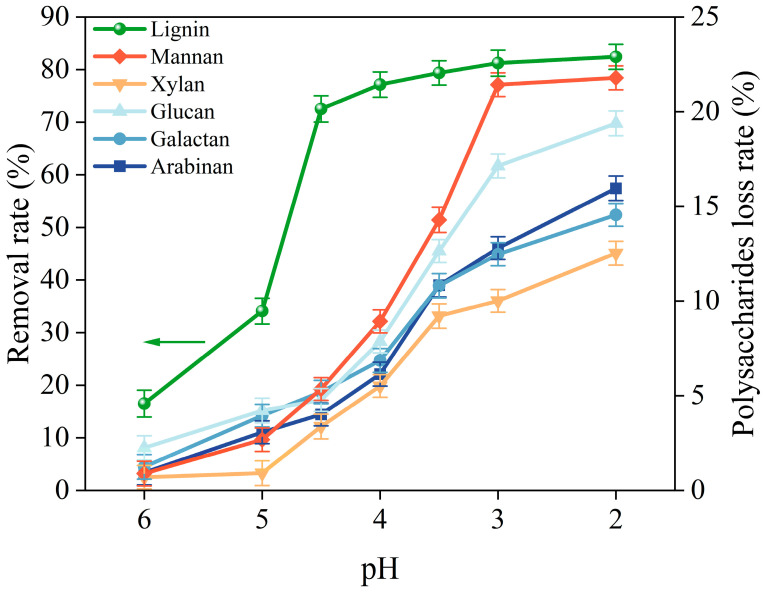
Effect of acid treatment on lignin and polysaccharides in pulping liquor.

As shown in Figure 4, the lignin removal rate increased significantly when the pH decreased to 4, followed by a minimal increase when the pH was further decreased to 2. The lignin removal rate reached 77.15% and 82.44% under the treatment of pH at 4 and 2, respectively. In addition, the polysaccharides in pulping liquor gradually decreased with the decrease in pH. During the acid treatment process of pulping liquor, the loss rate of mannan reached 21.79% at pH 2, followed by glucan and arabinan, with loss rates of 19.38% and 15.96%, respectively. It is noteworthy that the saccharide loss rate increased significantly after the pH decreased below 4. After acid treatment, the removal rate of lignin and loss rate of polysaccharides were 77.15% and 6.13%, respectively. In order to balance the recovery of polysaccharide resources and lignin removal efficiency, the optimal pH value of 4 was utilized for acid treatment followed by enzymatic hydrolysis of pulping liquor for XOS recovery.

### 2.3. Characterization of Recycled Lignin During Acid Treatment of the Pulping Liquor

**Figure 5:**
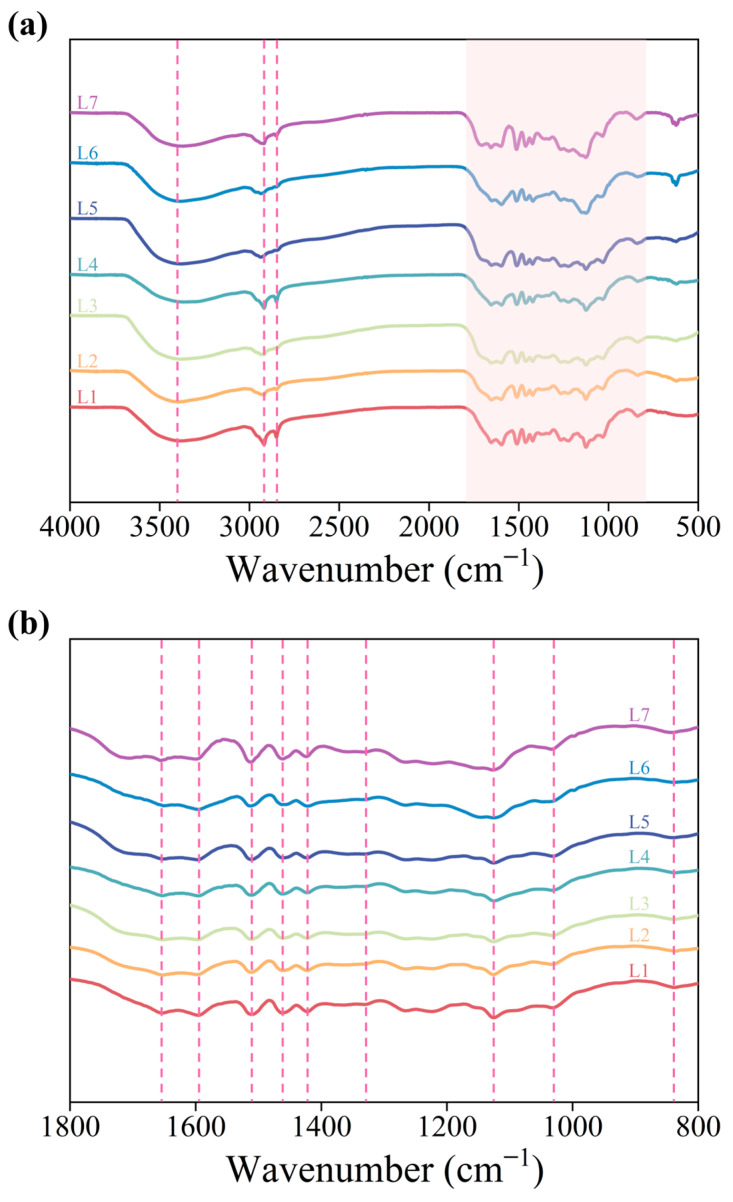
The FT-IR spectra of the sediments of L1−L7 recovered from the pulping liquor. (**a**) FT-IR spectra of lignin samples L1–L7. (**b**) Local amplified FT-IR spectra of lignin samples L1–L7.

In order to verify the chemical structure of lignin, FTIR was utilized to analyze the precipitated residue, as shown in Figure 5. The O-H stretching vibrations were found at 3420 cm^−1^, revealed by a wide absorption band [27]. The peaks at 2920 cm^−1^ and 2850 cm^−1^ can be assigned to the C-H stretching vibration absorption peaks of methyl and methylene groups. A weak telescopic vibrational absorption peak of lignin-conjugated carbonyl C=O was found at 1650 cm^−1^. Absorption peaks at 1595, 1510, 1460 and 1420 cm^−1^ are associated with C-H deformations related to aromatic ring vibrations as well as aromatic backbone vibrations [28]. The signal at 1330 cm^−1^ is attributed to the respiratory vibration of the condensation of syringyl (S) and guaiacyl (G) units [29]. Aromatic C-H in-plane deformation vibrations were detected at 1030 cm^−1^. A peak at 835 cm^−1^ denoted C-H out-of-plane deformation vibrations in positions 2 and 6 of S and all positions of the p-hydroxyphenyl (H) units [30]. The strength of the vibrational absorption peak at 1125 cm^−1^, which was assigned to S units, increased from L1 to L7, confirming that the high amount of S units in the residue was obtained under mild acid treatment [31,32]. It can be seen that the infrared spectrum curves of different lignin samples are similar, indicating that the structure of the main structure of lignin in the residue fraction does not significantly vary after acid treatment [33], and the abundant functional groups in the lignin indicate the potential application for the production of lignin-based high-value-added products [34,35].

**Figure 6:**
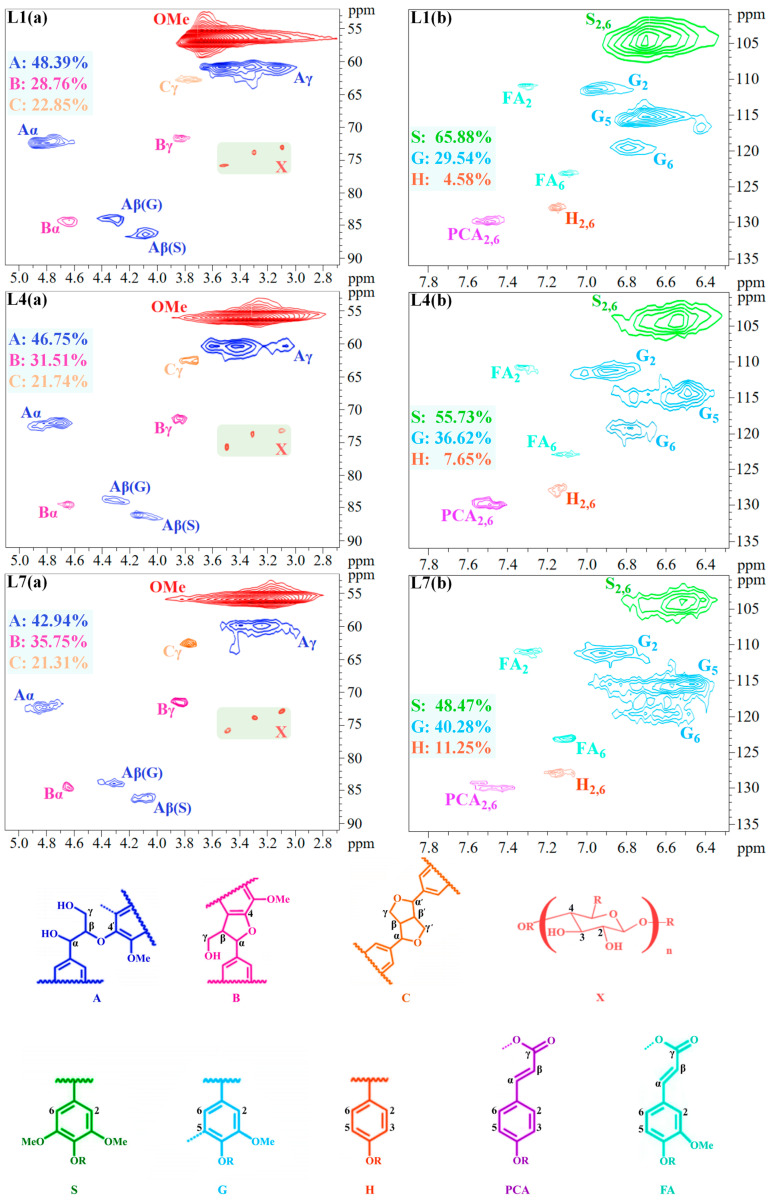
Two-dimensional HSQC NMR spectra of the L1, L4, and L7 and their main structures. (**a**) Structure of the aliphatic C-O region of lignin samples L1, L4 and L7. (**b**) Structure of the aromatic region of lignin samples L1, L4 and L7.

Figure 6 illustrates 2D HSQC spectra of the chemical structure of the precipitated lignin samples of L1, L4, and L7. The spectra of lignin chemical structures have two characteristic separate regions: the aliphatic C-O region (δC/δH: 50.0–95.0/2.50–6.00) and the aromatic region (δC/δH: 95.0–150.0/5.50–8.00) [36]. In the aliphatic region, the significant cross signal at δC/δH: 56.0/3.70 ppm is a characteristic signal of the methoxy group of lignin. Typical cross signals were observed at δC/δH: 60.2/3.40 ppm (Cγ-Hγ, Aγ), δC/δH: 72.3/4.83 ppm (Cα-Hα, Aα), δC/δH: 83.9/4.32 ppm (Cβ-Hβ, Aβ-G) and δC/δH: 86.2/4.11 ppm (Cβ-Hβ, Aβ-S), which verified the presence of the β-O-4′ substructure (A unit). The signals at δC/δH: 84.6/4.64 ppm (Cα-Hα, Bα) and δC/δH: 71.6/3.83 ppm (Cγ-Hγ, Bγ) indicate the presence of the phenyl β-5′ structure (B unit). The cross signal at δC/δH: 62.7/3.78 ppm (Cγ-Hγ, Cγ) confirm the presence of the resinoid alcohol β-β’ substructure (C unit). Structural signals of aryl rings of S, G and H were clearly detected in the aryl ring region of the precipitated lignin samples. The cross signals at δC/δH: 104.3/6.68 ppm (C2,6-H2,6, S2,6) belong to the S-type units. δC/δH: 111.4/6.95 ppm (C2-H2, G2), 114.8/6.71 ppm (C5-H5, G5) and 119.4/6.81 ppm (C6-H6, G6) are assigned to the G-type units. δC/δH: 128.1/7.16 ppm (C2,6-H2,6, H2,6) is attributed to the H-type units. The proportion of S-type units decreased incrementally with the decrease in pH, whilst the G and H ratio were increased (Figure 5). The results were in agreement with the FT-IR results. The precipitation of lignin could be related to the non-covalent π–π interactions between the phenyl rings in the decrease in S > G >H units [37]. Therefore, the high S units proportion existing in lignin fraction L1 and L4 were found, verifying that acid treatment of the pulping liquor with pH over 4 is more efficient. In addition, the presence of xylan moiety (X) was observed at δC/δH: 73.0/3.10 ppm (C2-H2, X2), δC/δH: 74.0/3.30 ppm (C3-H3, X3) and δC/δH: 75.7/3.50 ppm (C4-H4, X4). Meanwhile, cross signals of ferulic acid ester (FA) groups were detected at δC/δH: 111.1/7.30 ppm (C2-H2, FA2) and δC/δH: 123.1/7.12 ppm (C6-H6, FA6), and ferulic acid esters can form LCC with hemicellulose through ester and ether bonds in herbaceous plants [38]. These results confirmed the existence of LCC during the acid precipitation process, which is in agreement with the results of the saccharide loss during acid treatment.

Figure 6 displays the relative abundance of major inter-unit linkages in the lignin samples by a semi-quantitative HSQC NMR analysis. The predominant linkages in lignin samples are β-O-4′, followed by β-5′ and β-β′. It can be seen that with the decrease in pH, the proportion of β-O-4′ linkages in lignin samples significantly decreases. The β-O-4′ linkages in L7 (42.94%) and L4 (46.75%) were less than that in L1 (48.39%), implying that acid catalyzed depolymerization, and condensation of lignin subunits induced the structural variation in the major linkages [39]. The significant β-O-4′ linkages in L4 demonstrated less condensation of the lignin subunits than that of lignin in L7, indicating the potential application of lignin for functional materials.

In addition to the chemical structure, the weight-average molecular weight, number-average molecular weight and polydispersity index of lignin were measured by gel permeation chromatography (GPC). Table 2 showed the weight-average molecular weight, number-average molecular weight and polydispersity index of lignin residue in different fractions after acid treatment. As the pH decreased, Mw decreased from 3031 to 2128 g/mol and Mn decreased from 1588 to 1354 g/mol, indicating that lignin fractions with higher molecular weights are prone to precipitate first due to stronger π-π interactions induced by acid treatment. It has been shown that low-molecular-weight lignin typically contains more hydrophilic groups (e.g., hydroxyl (Ar-OH) and carboxyl (-COOH)) and therefore requires higher acid concentrations to reduce the charge effect and lead to lignin deposition [40,41]. As can be seen from Table 2, the dispersibility of lignin decreases during the gradual decrease in pH, and the polydispersity is relatively lower than most reported lignin by-products produced from the chemical pulping process. The low polydispersity index of the lignin fraction also indicated that the high heterogeneity of the recovered lignin is favorable for the utilization of lignin in the polymer industry [42].

### 2.4. Enzymatic Hydrolysis of Puling Liquor for the Recovery of XOSs

**Figure 7:**
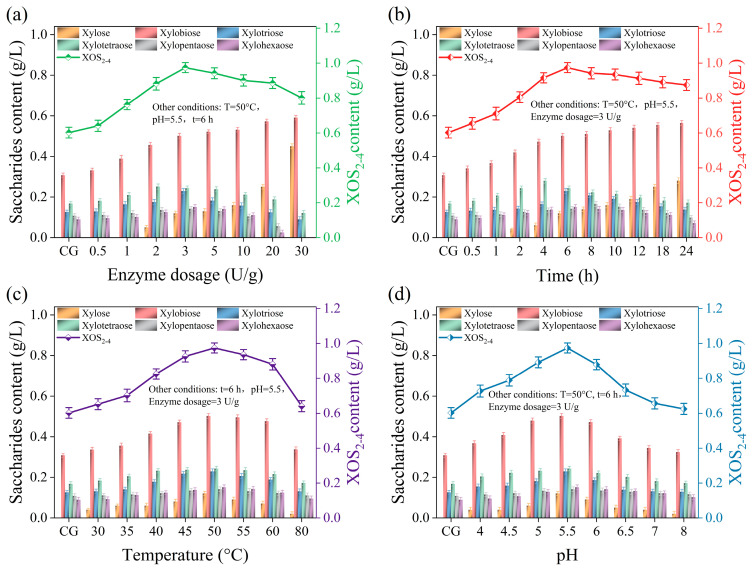
The effect of enzymatic hydrolysis conditions on the XOS content in acid treatment supernatant. (**a**) The effect of xylanase dosage on the XOS content in acid treatment supernatant. (**b**) The effect of enzymatic hydrolysis time on the XOS content in acid treatment supernatant. (**c**) The effect of enzymatic hydrolysis temperature on the XOS content in acid treatment supernatant. (**d**) The effect of enzymatic hydrolysis pH value on the XOS content in acid treatment supernatant.

The supernatant of the pulping liquor is rich in hemicellulose fraction, and xylan is the main component of hemicellulose in wheat straw [43]. Therefore, the supernatant was further hydrolyzed with xylanase to obtain the functional by-product of XOSs. From Figure 7a,b, it can be seen that with the increase in xylanase dosage or the extension of treatment time, the content of xylose and xylobiose in the acidification solution shows a gradual increasing trend, while the content of xylotriose, xylotetraose, xylopentaose, and xylohexaose first increases and then decreases; this was also the same for the content of XOS_2–4_. This is because when the enzyme dosage is low, there are fewer reaction sites provided by the enzyme. As the enzyme dosage increases, the number of enzyme molecules in the acid treatment solution increases, and the probability of enzyme molecules binding to the reaction substrate increases [44]. The substrate fully binds to the enzyme, and thus the higher XOS content was obtained with the elevated reaction rate. Continuing to increase the enzyme dosage induced the reduction in the degree of polymerization and xylose content. As the reaction time prolongs, the remaining enzyme molecules in the liquor after acid treatment further act on the glycosidic bonds of XOSs to form xylose with a lower degree of polymerization, leading to the increase in xylose content [45].

As shown in Figure 7c, temperature is an important factor affecting the effectiveness of enzyme treatment. The optimized temperature of 50 °C obtained a total XOS_2–4_ content of 0.97 g/L. As shown in Figure 7d, with the continuous increase in the pH value, the XOS_2–4_ content shows a trend of first increasing and then decreasing. This is because the pH value can change the conformation of xylanase and thus affect the binding between the enzyme’s active site and substrate [46,47]. It is found that the optimum pH of the enzyme treatment of the hemicellulose fraction is 5.5, and the high pH treatment of the pulping liquor (pH = 4) in the first stage allowed for less acid addition to obtain optimum enzymatic efficiency.

The content of xylose and XOSs in the hydrolysate is shown in Table 3. After treatment with xylanase, the content of xylobiose, xylotriose, and xylotetraose in the acidified solution increased by 61.29%, 76.92%, and 41.18%, respectively. The content of XOS_2–4_ increased from 0.61 g/L to 0.97 g/L, which was 59.02% higher than that before enzymatic hydrolysis. The recovery rate of XOS_2–4_ reached 51.87%. It can be concluded that xylanase treatment is an effective method for recovering XOSs from pulping liquor.

### 2.5. Mass Balance of the Extensive Utilization of Wheat Straw Based on Chemical–Mechanical Process

**Figure 8:**
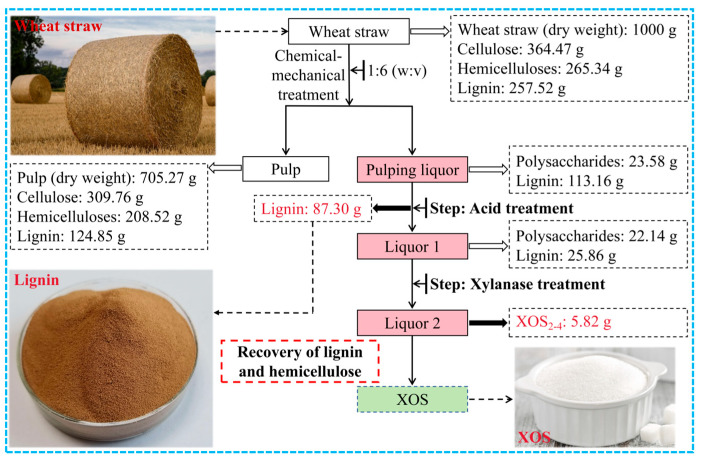
Mass balance of chemical–mechanical pulping process of wheat straw.

The mass balance of the utilization process of wheat straw is shown in Figure 8. After chemical and mechanical treatment, the yield of the pulp slurry is 70.53%. The total polysaccharides and the lignin content in the pulping liquor are 23.58 g and 113.16 g, respectively. The utilization rates of cellulose, hemicellulose, and lignin in the whole process reached 85.73%, 85.91%, and 82.38%, respectively.

The acid treatment coupled with the enzymatic treatment process can not only recover lignin from papermaking pulping liquor, but can also realize the utilization of hemicelluloses in the pulping liquor. Additionally, the separated lignin can be used to produce various high-value-added products, and XOSs can be used as functional food additives or feed additives [48], bringing additional profit to enterprises and facilitating the extensive utilization of non-woody biomass resources [49].

## 3. Materials and Methods

### 3.1. Materials

The pulping liquor of the wheat straw chemical–mechanical pulp was supplied by a paper mill in Shandong Province, China. Concentrated sulphuric acid (98%) was purchased from Yantai Far East Fine Chemical Co. (Yantai, China). Xylanase (enzyme activity 50,000 U) was purchased from Shandong Longkete Enzyme Preparation Co., Ltd. (Jinan, China).

### 3.2. Acid Treatment Pulping Liquor

A total of 80 g of pulping liquor was adjusted to a pH of 6 by adding 72% concentrated sulfuric acid solution with magnetic stirring at 200 rpm for 120 min. After that, the beaker was centrifuged at 10,000 rpm for 10 min, and the supernatant obtained from centrifugation was collected and preserved. The sediment obtained from centrifugation was collected and washed with distilled water, then centrifugation was continued, the washing was repeated four times, and the precipitate was collected followed by freeze-drying, which is denoted as L1. The pulping liquor was treated with 72% concentrated sulfuric acid solution to specified pH (5–2) following the abovementioned procedure, and the sediments were denoted as L2–L7, respectively (Table 4).

### 3.3. Xylanase Treatment

Firstly, the pH of 100 mL of acid treated supernatant was adjusted to the target pH using HCl/ NaOH solution, and then xylanase was added to the solution in a shaker (ZQZY-78AE, Zhichu, China) under different temperatures at 180 rpm. The enzyme dosage (0.5, 1, 2, 3, 5, 10, 20, 30 U/g), treatment time (0.5, 1, 2, 4, 6, 8, 10, 12, 18, 24 h), treatment temperature (30, 35, 40, 45, 50, 55, 60, 80 °C), and pH value (4, 4.5, 5, 5.5, 6, 6.5, 7, 8) were optimized. After the reaction is completed, the solution was quenched in a boiling water bath for 10 min to inactivate the xylanase, then it was centrifuged at 6000 rpm for 5 min. The supernatant was collected for further analysis.

### 3.4. Characterization

#### 3.4.1. Determination of Saccharides in the Pulping Liquor of Wheat Straw Chemical–Mechanical Pulp

The sugar analysis followed the previous NREL standard procedure [50]. A total of 5 mL of pulping liquor was hydrolyzed under 72% concentrated sulfuric acid in an oil bath for 60 min at 121 °C [51]. The supernatant was diluted and analyzed by ICS-5000 ion (ICS-5000 + , Thermo Fisher Scientific, Waltham, MA, USA) chromatography with CarboPacPA20 (3 mm × 150 mm). The injection volume was 25 μL, the column temperature was 30 °C, and the mobile phase was a gradient elution of 250 mM/L sodium hydroxide and distilled water at a flow rate of 0.4 mL/min. The concentration of monosaccharides before and after acid digestion can be measured using the above method, and the content of oligosaccharides is calculated from the increase in monosaccharides after acid digestion.

#### 3.4.2. Determination of Acetic Acid, Furfural and 5-HMF in Pulping Liquor

A total of 1 mL of pulping liquor was diluted 30 times and analyzed on an high-performance liquid chromatography (1260 II, Agilent, Santa Clara, CA, USA) with a Bio-Rad Aminex HPX-87H (300 × 7.8 mm) (Aminex HPX-87H, Bio-Rad, Hercules, CA, USA) column at a detection wavelength of 210 nm with an injection volume of 25 μL, a column temperature of 50 °C, and a mobile phase of 5 mmol/L sulfuric acid at a flow rate of 0.6 mL/min. The contents of acetic acid, furfural and 5-HMF in the pulping liquor were determined with the internal standard method.

#### 3.4.3. Lignin Characterization

All the lignin samples were characterized by the FTIR, 2D-HSQC, GPC, etc. The lignin content of the samples was determined according to TAPPI UM 250 using UV spectrophotometry (T9S, PERSEE, Beijing, China). Use Malvern Zetasizer to determine the Zeta potential and particle size of the samples (Nano-ZS90, Malvern, UK). The FTIR measurement was performed on a spectrophotometer (Invenios, Bruker, Bremen, Germany). The samples were scanned with a wavenumber range from 4000 to 500 cm^−1^ at a resolution of 4 cm^−1^. For the 2D-HSQC NMR measurements, 40 mg lignin was dissolved in 0.6 mL DMSO-d6 and subsequently measured on a spectrometer (JNM-ECZL600G, JEOL, Kyoto, Japan). The weight-average molecular weight (M_w_), number-average molecular weight (M_n_), and polydispersity (M_w_/M_n_) of the lignin samples were performed on a gel permeation chromatography (GPC) system (Waters E2695, Waters, Williamsburg, VA, USA) after acetylation. Polystyrene was used for calibration.

## 4. Conclusions

This study provides a facile and low-cost process for the recovery of lignin and hemicellulose from the pulping liquor of wheat straw chemical–mechanical pulp, and hemicelluloses were further upgraded to XOSs via enzymatic hydrolysis. The negative charged ionic moieties (carboxylic and phenolic groups) present in the lignin of the pulping liquor are deprotonated with acid treatment, and the lignin-degraded products would further aggregate via π-π interaction to form lignin clusters, leading to the separation of lignin from the pulping liquor. The decrease in pH treatment-induced sugar loss is due to the presence of LCC, and lignin undergoes depolymerization and condensation during acid treatment, which is confirmed by FTIR and NMR analysis. By leveraging lignin removal and the sugar loss rate, the mild acid treatment coupled with the enzymatic process was realized as an efficient lignin removal of 77.15% from the pulping liquor at a mild pH of 4. Then, the pulping liquor was further treated with xylanase to obtain XOSs, and the optimized enzymatic conditions of an additional amount, pH, temperature and time were 3 U/g, 5.5, 50 °C and 6 h, respectively. Additionally, the xylanase treatment of pulping liquor attained a recovery rate of 51.87% for XOSs. The mild acid treatment coupled with the enzymatic hydrolysis process realized an efficient recovery of lignin and hemicelluloses, providing a new route for the comprehensive utilization of non-woody biomass materials in the practical pulping industry.

## Figures and Tables

**Table 1:** Main components in the pulping liquor prepared under acid treatment process.

Samples	Concentration (g/L)								
	Arabinan	Galactan	Glucan	Xylan	Mannan	Acetic Acid	Furfural	5-HMF	Lignin
Pulping liquor	1.17 ± 0.11	0.48 ± 0.01	0.36 ± 0.01	1.87 ± 0.13	0.06 ± 0.01	3.26 ± 0.17	0.38 ± 0.02	0.11 ± 0.01	18.86 ± 0.33
pH 6	1.16 ± 0.11	0.48 ± 0.01	0.35 ± 0.01	1.86 ± 0.14	0.06 ± 0.00	3.31 ± 0.14	0.38 ± 0.01	0.11 ± 0.00	15.75 ± 0.32
pH 5	1.14 ± 0.12	0.46 ± 0.02	0.34 ± 0.02	1.85 ± 0.14	0.06 ± 0.01	3.37 ± 0.13	0.38 ± 0.00	0.11 ± 0.01	12.43 ± 0.31
pH 4.5	1.13 ± 0.11	0.46 ± 0.01	0.34 ± 0.01	1.81 ± 0.12	0.05 ± 0.00	3.42 ± 0.14	0.38 ± 0.02	0.11 ± 0.00	5.18 ± 0.22
pH 4	1.10 ± 0.11	0.45 ± 0.01	0.33 ± 0.01	1.77 ± 0.13	0.05 ± 0.00	3.59 ± 0.12	0.38 ± 0.01	0.11 ± 0.02	4.31 ± 0.22
pH 3.5	1.05 ± 0.11	0.43 ± 0.02	0.31 ± 0.01	1.70 ± 0.12	0.05 ± 0.01	3.71 ± 0.13	0.38 ± 0.00	0.11 ± 0.01	3.89 ± 0.16
pH 3	1.02 ± 0.09	0.42 ± 0.01	0.30 ± 0.00	1.68 ± 0.13	0.04 ± 0.01	3.75 ± 0.13	0.39 ± 0.02	0.10 ± 0.00	3.54 ± 0.14
pH 2	0.99 ± 0.07	0.41 ± 0.01	0.29 ± 0.00	1.63 ± 0.12	0.04 ± 0.00	3.81 ± 0.14	0.39 ± 0.01	0.10 ± 0.01	3.31 ± 0.14

**Table 2:** The weight-average molecular weight (M_w_), number-average molecular weight (M_n_), and polydispersity index (PDI, M_w_/M_n_) of the lignin samples.

Samples	M_w_ (g/mol)	M_n_ (g/mol)	PDI
L1	3031	1588	1.91
L2	2897	1557	1.86
L3	2695	1482	1.82
L4	2496	1462	1.71
L5	2394	1427	1.68
L6	2283	1381	1.65
L7	2128	1354	1.57

**Table 3:** XOS content before and after enzymatic hydrolysis under optimal processing conditions.

Samples	Concentration (g/L)				
	Xylose	Xylobiose	Xylotriose	Xylotetraose	XOS_2–4_
Before enzymatic hydrolysis	0	0.31 ± 0.02	0.13 ± 0.01	0.17 ± 0.01	0.61 ± 0.02
After enzymatic hydrolysis	0.12 ± 0.01	0.50 ± 0.01	0.23 ± 0.01	0.24 ± 0.01	0.97 ± 0.03

**Table 4:** The target treatment pH of corresponding samples.

Samples	Target pH Value
L1	6
L2	5
L3	4.5
L4	4
L5	3.5
L6	3
L7	2

## Data Availability

The datasets generated for this study are available upon reasonable request from the corresponding author.
